# Mercury Contamination as an Indicator of Fish Species’ Trophic Position in the Middle Araguaia River, Brazil

**DOI:** 10.3390/toxics11110886

**Published:** 2023-10-29

**Authors:** Lilian de Castro Moraes, José Vicente Elias Bernardi, João Pedro Rudrigues de Souza, Joelma Ferreira Portela, Hasley Rodrigo Pereira, Hugo de Oliveira Barbosa, Nayara Luiz Pires, Lucas Cabrera Monteiro, Ygor Oliveira Sarmento Rodrigues, Ludgero Cardoso Galli Vieira, Carlos José Sousa Passos, Jurandir Rodrigues de Souza, Wanderley Rodrigues Bastos, José Garrofe Dórea

**Affiliations:** 1 Programa de Pós-Graduação em Ciências Ambientais, Faculdade UnB Planaltina, Universidade de Brasília, Planaltina 73345-010, DF, Brazil; lilian.moraes@gmail.com (L.d.C.M.); hasleybio08@gmail.com (H.R.P.); hugobioueg@gmail.com (H.d.O.B.); nayara_luiz@hotmail.com (N.L.P.); ygorsarmento@gmail.com (Y.O.S.R.); 2 Laboratório de Geoestatística e Geodésia, Faculdade UnB Planaltina, Universidade de Brasília, Planaltina 73345-010, DF, Brazil; 3 Laboratório de Química Analítica e Ambiental, Instituto de Química, Universidade de Brasília, Brasília 70919-970, DF, Brazil; joaoprsouza@outlook.com (J.P.R.d.S.); portela.joelma@gmail.com (J.F.P.); rodsouza@unb.br (J.R.d.S.); 4 Programa de Pós-Graduação em Ecologia, Instituto de Ciências Biológicas, Universidade de Brasília, Brasília 70910-900, DF, Brazil; lcabreramonteiro@gmail.com; 5 Núcleo de Estudos e Pesquisas Ambientais e Limnológicas, Faculdade UnB Planaltina, Universidade de Brasília, Planaltina 73345-010, DF, Brazil; ludgero@unb.br; 6 Faculdade UnB Planaltina, Universidade de Brasília, Brasília 73345-010, DF, Brazil; cjpassos@unb.br; 7 Laboratório de Biogeoquímica Ambiental, Universidade Federal de Rondônia, Porto Velho 76901-000, RO, Brazil; bastoswr@unir.br; 8 Faculdade de Ciências da Saúde, Universidade de Brasília, Brasília 70919-970, DF, Brazil; jg.dorea@gmail.com

**Keywords:** food chain, food web, bioaccumulation, sediment

## Abstract

This study evaluates the use of mercury (Hg) concentrations in fish muscle tissue to determine a species’ trophic position (TP) in its environment. A campaign conducted in 2019 along 375 km in the middle Araguaia River basin, Brazil, resulted in 239 organisms from 20 species collected. The highest total mercury (THg) concentrations were found in *Pellonacastelnaeana* (6.93 µg·g^−1^, wet weight) and in *Triportheus elongatus* (3.18 µg·g^−1^, wet weight), whose TPs were different according to the FishBase database. However, they occupied the same trophic level in this study. The intra-specific comparison showed a difference in Hg concentrations between individuals captured in distinct sites. The study of the biota–sediment accumulation factor (BSAF) showed that spatiality interferes with a species’ TP. Statistical analyses revealed that when we used a predicted species’ TP based on each individual’s size, it explained 72% of the variability in THg concentration across all fish species. Multiple regression analysis confirmed that standard length and FishBase values are positively associated with THg (R^2^ = 0.943). These results point to Hg as a viable indicator of a fish species’ TP since it reflects regional, biological, and environmental factors, as demonstrated here for the middle Araguaia River.

## 1. Introduction

Trophic interactions within food webs can strongly influence the pathways and efficiencies of material and energy transfer to higher-level consumers, which collectively determine the net-integrated “trophic positions (TP)” of species [1]. To date, one of the most used sources of quantitative information on the trophic position (TP hereafter) of fish species is the web-based relational database FishBase [2]. The development of this database was a collaboration between the WorldFish Center and the Food and Agriculture Organization of the United Nations (FAO), with support from the European Commission (EC) [3]. FishBase began cataloging key life history parameters of the world’s fish species [2] and became an important online resource that contains information on over 34,000 fish species (R. Froese, FishBase, personal communication).

The FishBase uses diet composition data to estimate feeding habits and thus identify a fish species’ TP [2]. The model used in FishBase to calculate TP represents a mean of the species across previously published studies [4]; however, populations have inherent specific differences in life history traits [5], and thus, parameters derived from geographically distant populations may be less precise for local applications than locally derived parameters. Mean TP values averaged over time and area may conceal potentially high variability associated with foodweb dynamics and ontogenetic changes. TP should display spatio-temporal variations according to fish age or predator total length [6]. For instance, the trophic position of larger consumers is expected to be lower in tropical than temperate regions to compensate for energy limitation [7]. In addition, the variety of sources and differing quality of information in FishBase may allow biases to occur [8]. Furthermore, Hussey et al. found through isotope studies that the mean TP (FishBase) may not represent the best estimated trophic position for fish species, particularly large predators [9].

Since it is important to consider local variations to determine a fish species’ TP, the presence and concentration of mercury (Hg) in fish play a crucial role in this context. Mercury (Hg) is a toxic metal that is naturally present in the environment and can enter trophic webs in its organic form (methylmercury). Within an organism, this metal adheres to organic tissue and bioaccumulates, which leads to biomagnification across the trophic web. Thus, organisms at the end of the food web should have a higher level of Hg in their tissues. This could affect humans and lead to exposure to mercury since they consume fish, especially muscular tissue [10].

Due to this ability to bioaccumulate in an organism and biomagnify across the trophic web, Hg presence and concentration in fish could be a useful functional indicator of fish species’ TP. The main objective of this study is to assess if Hg contamination could be used to determine the TP of fish species. To this end, we measured total mercury concentrations (THg) in muscle tissues of 20 different fish species from the Araguaia River, a large river in the Cerrado, aiming to relate these concentrations to fish size and compare our results with information on trophic positions from the FishBase. In general, trophic positions provided in this database are broad estimates unrelated to spatiality or seasonality [3]. Here, they were used as reference values and compared using linear regression to other variables for each species. We also determined the biota–sediment accumulation factor (BSAF) for each individual to show that spatiality is a driver of fish Hg contamination and related it to the geology of the area and land use. In this paper, we combined the assessment of mercury concentrations in fish muscle tissue in relation to fish species’ standard length (SL), FishBase values for TP, BSAF, geological characteristics, and land use in a multivariate statistical analysis framework.

## 2. Materials and Methods

### 2.1. Study Area

**Figure 1:**
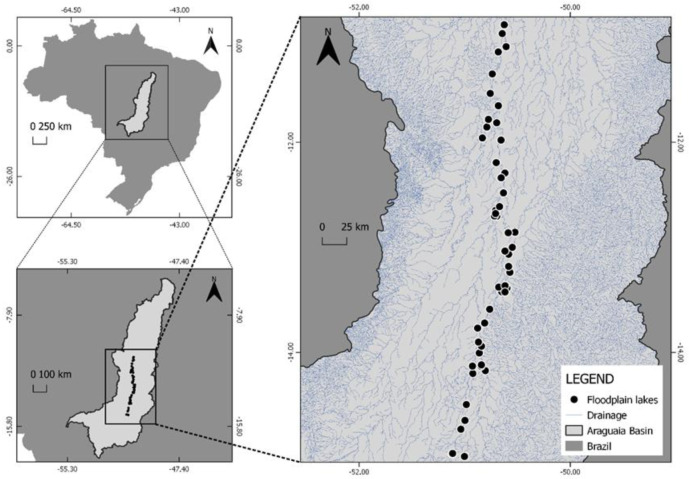
Location of the sampling points in the Araguaia River floodplain in January 2019.

This study was performed in the middle Araguaia River floodplain, which is located in the Tocantins–Araguaia watershed. The Araguaia is the main river draining the Brazilian Cerrado, with an area close to 377,000 km^2^ and a mean annual discharge of 6420 m^3^s^−1^ [11]. We collected samples in January 2019, in a hydrological period of flooding (November to April), mainly in lentic environments, at fifty different locations (P01 to P50). About 375 km was traveled along the river, including five tributaries and the island of Bananal in almost all its extension (Figure 1).

### 2.2. Sampling

Fish sampling was carried out using gill nets 10 m long with mesh openings of 15, 25, 30, 35, and 40 mm between nodes. The gill nets with different meshes were spliced together, forming a single net 50 m long. Thus, two sets of nets, as described previously, were placed in each lake during the day for approximately 40 min. We collected all fish specimens captured (*n* = 239). After taking their image, we measured each individual’s weight (g), standard length (cm), and total length (cm). Fish species were established according to taxonomy and identification keys [12,13], which were later confirmed by specialists. Fish feeding habits were taken from the FishBase [2]. A sample of each individual’s dorsal muscle was removed for analysis. Muscle samples were labeled, stored in transparent polyethylene bags, and kept frozen until analysis.

We collected bottom sediment samples manually or using the Eckman dredge in all sampling points. All samples were stored in polyethylene bags and kept cool until sample preparation and chemical analysis.

### 2.3. Sample Treatment and Mercury Determination

All glassware used for analyses was submitted to rigorous cleaning procedures that included acid washings (with HNO_3_ 5% for 24 h) and rinsing with ultrapure water. Sediment samples were dried in an oven at 40 °C, macerated, and subjected to a sieve shaker for 10 min, where they passed through 600 µm, 250 µm, 120 µm, and 20 µm granulometric analysis sieves (Bronzinox, Mestrino, Italy). The smallest particles, homogenized, were stored in Eppendorf tubes for the quantification of Hg. Fish muscle samples were defrosted and wet-weighed at the time of analysis.

Total mercury (THg) represents the sum of all mercury species present in the analyzed samples [14]. The THg concentrations in the solid samples were analyzed at the Analytical and Environmental Chemistry Laboratory (LQAA) at the University of Brasilia using 254 nm thermal decomposition–atomic absorption spectrometry (TDAAS) with a Zeeman correction for background absorption using a Zeeman RA 915+ analyzer (Lumex Instruments, Mission, BC, Canada). Quantifying Hg was made using external standard calibration with diluted solutions of a Hg 1000 mg·L^−1^ standard solution (Aldrich, St. Louis, MO, USA). The reference material, standard solutions, blanks (ultrapure water), and samples were analyzed in triplicates. Analytical quality control was performed using certified reference materials (CRMs) BCR-463 and DORM1 for fish and SS-2 for sediments. The average recovery of CRMs varied from 108% to 120% for fish and reached 88% for sediments. The coefficient of variation between replicates of fish samples varied from 0 to 15% and between replicates of sediments from 0 to 16%. The detection limits for THg were 0.06 ng (fish) and 0.024 ng (sediments).

### 2.4. Biota–Sediment Accumulation Factor

The biota–sediment accumulation factor (BSAF) expresses the net bioaccumulation of chemicals by an organism as a result of absorption from all environmental sources and processes [15]. BSAF is found through the ratio between the concentration of the contaminant in the biota and its concentration in the sediment [16]. We calculated the BSAF for each fish.

$$\mathrm{BSAF}=\frac{\left[\mathrm{Hg}\right]\mathrm{Biota}}{\left[\mathrm{Hg}\right]\mathrm{Sediment}}$$

To examine the relationship between geological groups, land use, and the BSAF, we assigned each sampling point to a geological terrain and one of two land uses. Sampling points being closer than twelve kilometers from an urban area were labeled “Urban” and those being farther than twelve kilometers were labeled “Not Urban”. Based on geological contact and outcrops, we divided the sampled area into three groups: NPx (Neoproterozoic), NPx/Qag/Pp (Neoproterozoic/Paleoproterozoic), and Qag (Quaternary).

### 2.5. Statistical Analyses

In order statistically to compare the THg concentrations in fish from different guilds, we used descriptive statistics to determine the mean values for the duplicates of each sample (analytical blanks, CRM, and fish muscle), as well as to determine the range of each parameter, standard deviation, and coefficient of variation for each analysis. All variables were subjected to the Shapiro–Wilk (*n* < 50) or Kolmogorov–Smirnov (*n* > 50) tests to assess the distribution of the data.

We did a series of linear regressions to identify the best variable to classify fish species more efficiently in trophic levels (*n* = 239 samples). We chose this method because linear regression models allow values to be predicted based on the relationship between a variable of interest (dependent variable) and one or a set of predictor variables (independent variables). In addition to predicting values, linear regression models provide metrics for assessing the strength of the prediction and the significance of the predicted values. The coefficient of determination (R^2^) was used to measure the proportion of the dependent variable explained by the dependent variables. We used the student’s *t*-test to assess the significance of the predictive equations of each linear regression, evaluating whether the linear regression models’ angular coefficients (b) are statistically different from zero. A *p*-value < 0.05 indicates that the models are statistically significant. Due to how FishBase values are calculated (it is a sum, therefore, logarithmized), we used the log standard length (SL) and natural logarithm (ln) values in the analysis. 

We performed an initial linear regression model to determine the relationship between FishBase values for trophic position (dependent variable) and SL (dependent variable). This analysis allowed us to infer that this relation is highly significant and provided us with predicted values for FishBase based on the SL. Thus, instead of the mean value provided by FishBase (categorical), we obtained continuous trophic-level values corrected based on the morphological characteristics of each individual (i.e., SL). We ran another linear regression between THg (dependent variable) and SL (independent variable). In order to check if THg could be used as a parameter to determine trophic position, we performed a linear regression between THg (dependent variable) and FishBase (independent variable). We performed a linear regression model to determine the significance between THg values and predicted values for FishBase (based on SL). We also carried out a multiple linear regression model to assess the linear relationship and significance of the interaction between SL and FishBase (independent variables) on the THg concentration distribution (dependent variable).

To examine the relationship between THg and the FishBase values for the trophic position, we determined three levels for FishBase values and assigned each individual to a level: from 2.0 to 2.9 (*n* = 123); from 3.0 to 3.9 (*n* = 93); and from 4.0 to 5.0 (*n* = 23). Considering that the variables had a normal distribution, the difference between the groups was assessed using a one-way analysis of variance (ANOVA) followed by the Tukey honestly significant difference (HSD) posthoc test to know more about the specific groups that had a significant effect on THg concentrations (pairwise comparison). We also classified the BSAF values according to each sampling site’s geological formation and land use. These subsets of data were not normally distributed and were therefore evaluated using non-parametric tests. The difference between the geological groups (Npx, Npx/Qag/Pp, and Qag) was assessed using the Kruskal–Wallis and Dunn’s posthoc tests, while the difference between the land use classes (Urban and Not Urban) was assessed using the Mann–Whitney Utest. We considered the probability *p* < 0.05 with significant differences and confidence intervals of 95% between averages for the tests performed. The statistical analyses were performed with XLSTAT software 2021.4.1.1182.

## 3. Results

This study presents total Hg concentrations in muscle tissues of twenty species of fish (Table 1). 

**Figure 2:**
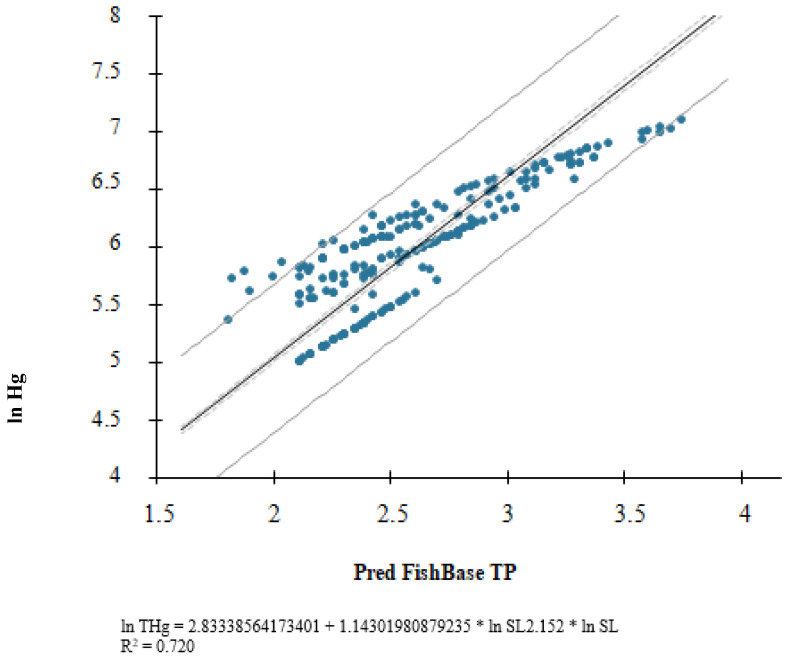
Scatterplot of the natural logarithm of predicted FishBase values (based on standard length) and THg concentrations in muscle tissue (*p* < 0.001) (*n* = 239).

**Figure 3:**
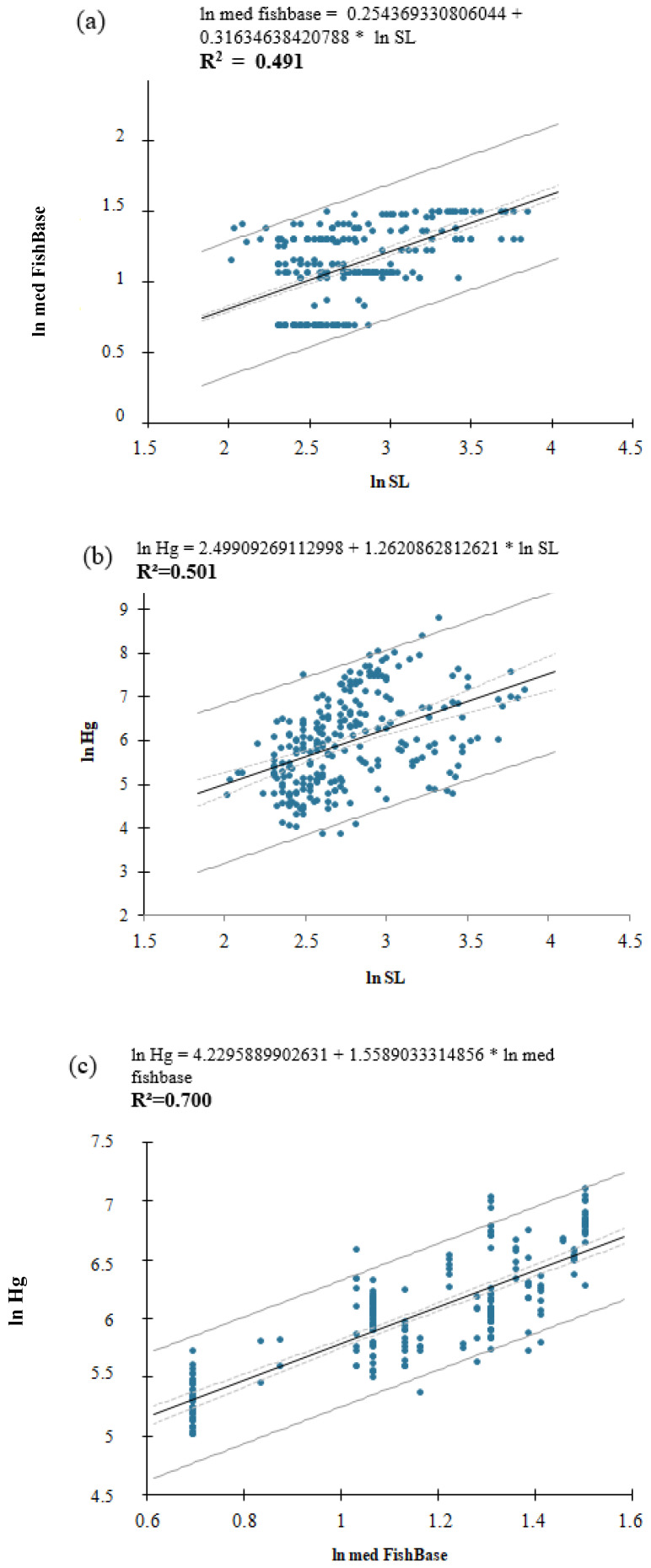
Scatterplots of the natural logarithm of (**a**) standard length (SL) and FishBase (*p* < 0.0001), (**b**) standard length and total Hg concentrations (*p* < 0.0001), and (**c**) FishBase and total Hg concentrations (*p* < 0.0001) (*n* = 239).

We confirmed that the THg concentration in fish muscle tissue could substitute FishBase values to determine a species’ trophic position (Figure 2) based on our initial statistical analyses (Figure 3). Regression of the mean THg concentration in muscle tissue and predicted values for FishBase based on the SL for all samples was positive and highly significant (R^2^ = 0.720; *p* < 0.001) and is shown in Figure 2. The high R^2^ value indicates that the trophic level predicted from each individual’s size is a significant descriptor for explaining the variability in the THg concentration in the Araguaia River basin fish. It alone explained 72% of the variability.

Figure 3 shows the results of the initial correlations between standard length (SL) and FishBase (3a); standard length (SL) and total Hg (THg) concentrations (3b); and THg and FishBase (3c). There was a strong and significant positive correlation between standard length (cm) and FishBase values for the trophic position (R^2^ = 0.491; *p* < 0.0001), between standard length and THg concentration in muscle tissue (R^2^ = 0.501; *p* < 0.0001) and between THg concentration in muscle tissue and FishBase values for the trophic position (R^2^ = 0.700; *p* < 0.0001).

**Figure 4:**
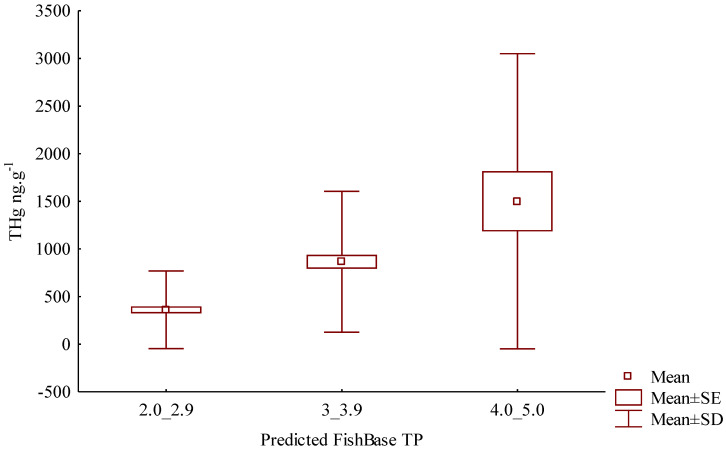
Boxplot showing total mercury (THg) variation in muscle tissue of fish that belong to different predicted FishBase categories. Tukey: a/b: *p* = 0.0000001; b/c: *p* = 0.0000001; and a/c: *p* = 0.0541245. TP: trophic position; SE: standard error; and SD: standard deviation.

Multiple regression analysis (Table 2) confirmed that standard length and FishBase values (trophic levels) are positively associated with THg (R^2^ = 0.943). We grouped individual fish according to the predicted values for FishBase based on the standard length: (a) between 2.0 and 2.9 (*n* = 123), (b) between 3.0 and 3.9 (*n* = 93), and (c) between 4.0 and 5.0 (*n* = 23) in order to compare the THg concentration in each group, which represents trophic levels. Univariate results (factorial ANOVA) showed that THg was significant within each category (*p* = 0.0000001). The multivariate analyses comparing THg between the categories revealed that the difference is significant (Figure 4). 

We considered the difference between groups a and c significant, and this analysis confirmed that THg concentration varies and increases as the trophic chain grows and reinforced that Hg can be used to determine a fish species’ trophic position. The THg concentration presented greater variability in category c, which contains species that have a carnivorous or piscivorous diet. This variability would have been even greater if individuals had reached their maximum standard length, but they did not.

We found the highest total mercury concentrations in two specimens of *Pellona castelnaeana* collected in two distinct sampling sites: 6.93 µg·g^−1^ and 4.54 µg·g^−1^, followed by two specimens of *Triportheus elongatus* captured in two distinct locations: 3.18 µg·g^−1^ and 3.08 µg·g^−1^. According to the FishBase (Table 1), they belong to different TPs (3.7 ± 0.56 and 2.9 ± 0.31, respectively), but our study revealed that both species occupy high trophic positions (TP) in their habitats.

**Figure 5:**
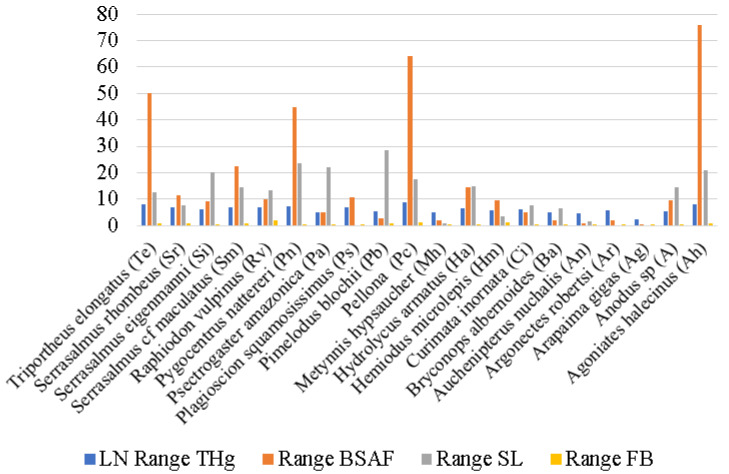
Vertical bar graph of the range of total Hg (THg) concentrations in muscle tissue (natural logarithm), the biota–sediment accumulation factor (BSAF), standard length (SL), and predicted FishBase values (based on standard length) (FB) (*n* = 239).

In order to confirm that spatiality interferes with a species’ TP, we calculated the BSAF for each individual fish and its range within each species. Figure 5 shows how the BSAF range varied in freshwater fish from the same species in different geographic locations. We observed wider ranges in *Agoniates halecinus* (piscivore; 75.77), which we collected in 16 sampling sites, followed by *Pellona castelnaeana* (carnivore; 64.18) in eight sampling sites, *Triportheus elongatus* (omnivore; 50.00) in 10 sampling sites, and *Pygocentrus nattereri* (piscivore; 44.92) in 19 sampling sites. Despite being omnivorous, *Triportheus elongatus* has a similar feeding habit to *Agoniates halecinus* (FishBase = 2.9). All four species had the highest THg concentrations, so they were considered top-of-the-chain.

**Figure 6:**
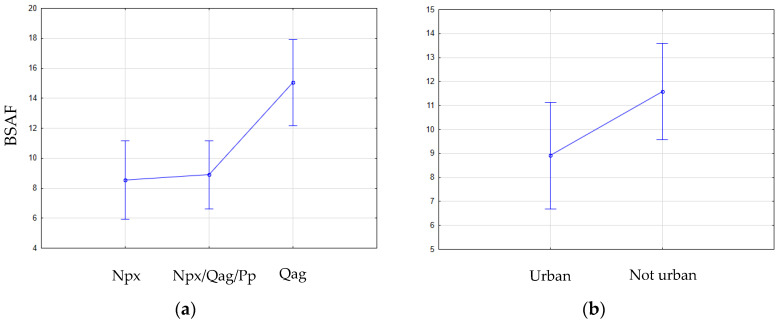
Difference in biota–sediment accumulation factor (BSAF) values according to (**a**) geological groups (*p* = 0.0058) and (**b**) proximity to urbanized areas (*p* = 0.0430).

Both geological formation (Kruskal–Wallis: *p* = 0.0058) and land use (U test: *p* = 0.0430) had a significant influence on BSAF values (Figure 6). The BSAF was significantly higher in Qag terrains and sites distant from urbanized areas.

## 4. Discussion

Although our data did not encompass the entire Rio Araguaia food web, the THg and standard length indicated a wide range of trophic positions. Across all analyzed fish species, predicted trophic levels based on each individual’s size explained 72% of the variability in the THg concentration in fish of the Araguaia River basin (Figure 2). The TP-Hg plot shows remarkably consistent distributions and suggests that high Hg concentrations can be expected as TP FishBase predicted values increase. This confirmed our hypothesis that mercury concentration could substitute FishBase values to determine a fish species’ trophic position. Moreover, it takes into consideration the wide diversity of species’ morphologies, life-history strategies, and habitat characteristics. Since fish acquire Hg from feeding, this finding suggests that the same fish species may occupy different TPs in distinct ecosystems, and our results showed that THg contamination is a more precise tool to establish a fish species’ TP in its habitat.

Our findings that standard length (cm) and FishBase values for trophic position were strongly correlated are in agreement with research carried out in other parts of the globe. Dantas et al. compared freshwater and marine environments in tropical and temperate climates and found that the fish trophic position increased with body size for both climates and ecosystems [7]. Romanuk, Hayward, and Hutchings analyzed 8361 fish species and observed a positive correlation between length and trophic position across all omnivorous and carnivorous fish species [17]. Although the increase in energy demand with body size and the reduced availability of energy at higher trophic positions may promote a negative correlation between trophic position and body size [18,19], this correlation was positive in the tropical environment of the Araguaia River.

Bastos et al. [20], who studied fish from the Madeira River (Amazon), also described a positive linear relationship between standard length and THg concentration in muscle tissue. On the other hand, also in the Amazon, Matos et al. did not detect such a correlation in omnivorous species [21]. This may be due to other mechanisms operating to counteract energetic limitation [17]. Growth rate, for example, is an important factor. Different species might have similar sizes and trophic positions but distinct growth rates. Fast growth contributes to low THg, whilst slow growth favors high THg [22].

Since the fish size measured as length or weight provides an index of age, and high trophic position (TP) and age are primary reasons for high concentrations of mercury in fish [22], our study corroborated this by showing a strong and significant correlation between THg concentration in fish muscle tissue and FishBase values for the trophic position. Hussey et al. and Kwon et al. also found a positive relation between THg concentrations and trophic levels [9,23]. The feeding habit contributes to variations of THg concentrations as well as the trophic position. Thus, species found at the top of the trophic web, essentially carnivorous, will present higher concentrations in relation to species near the base [24].

Our study included a multiple regression analysis (Table 2) whose *p*-value < 0.0001 showed a statistically significant association between standard length, FishBase values, and the THg in fish muscle, which confirmed that these variables are strongly associated with THg and explained 94% of its variation.

We certified that spatiality interferes with a species’ TP (Figure 5) since their diet will vary according to what is available to them. *Triportheus elongatus* is an omnivorous fish species that feeds on plants, detritus, and other animals [25]. According to the FishBase, its TP is 2.9. However, when we predicted the trophic level based on each individual’s size, eight specimens would belong to group a (FishBase 2.0–2.9) and seven to group b (FishBase 3.0–3.9) (Figure 4). These species’ BSAF range (Figure 5) was high, which indicates that these individuals were accumulating Hg differently. It is noteworthy that some species of the genus *Triportheus* constitute an important element in both commercial and subsistence fisheries [26]. In the Amazon basin, *Triportheus* is among the fish most often consumed by humans [27]. Other species that had high ranges of the BSAF, such as *Pellona castelnaeana*, *Agoniates halecinus*, and *Pygocentrus nattereri*, also had individuals present in more than one category (Figure 4). They were present in diverse sampling sites and displayed distinct Hg concentrations. Divergent sizes, distinct life phases and/or different types of diet (generalist, specialist, and opportunist) may contribute to the large dispersion of Hg concentration values relative to trophic position [24]. 

The range in the biota–sediment accumulation factor for freshwater fish specimens shows spatial variability in a species’ THg accumulation. This may be due to the distribution of Hg between the sediment and the water column, the food web length, the bioavailability of the contaminant, and the rates of metabolic transformation of the chemical in the food web, which vary between locations [28]. The BSAF is a specific measurement of the sampling point and represents its actual conditions [15]. The feeding habit and habitat may affect the extent of contaminant bioaccumulation [29]; therefore, the fact that mercury bioaccumulates in an organism [30] makes it possible that food availability and size of the food chain play an important role in how much Hg a fish species presents.

We did not locate a clear source of Hg in the study area, but we considered that the amount of Hg in the environment may depend on its geological characterization [31], land use, and proximity to urban areas [32]. We found that in the middle Araguaia floodplain, geological formation and proximity to urbanized areas significantly impacted BSAF values, being higher in Qag terrains and non-urban areas. This result was expected since the BSAF is a ratio, and the higher the THg concentration, the lower the BSAF will be. Ioele et al. and Tong et al. concluded that urbanization impacts mercury content in the aquatic environment; therefore, it should be lower in non-urban areas [33,34]. Rocks that form the Quaternary terrains (Qag) are mainly sedimentary, which do not present a metal binding capacity [35], thus explaining why its BSAF is higher than in terrains of mafic and volcanic rocks.

The presence of mercury in fish from the Araguaia River is worrisome because fish are part of the diet of the local population, and many fish individuals showed mercury concentrations above the exposure limits accepted by different environmental agencies. Thus, identifying the sources of this hazardous metal, which leads to its buildup in specific locations, is a crucial initial stride toward formulating effective emission control strategies and targeting polluted areas for remediation. Innovative and encouraging methods have emerged, such as employing environmental forensics tools for in-depth source-attribution studies. Stable isotopes of heavy metals (such as copper, lithium, and zinc) have seen a growing application in this regard [36,37,38,39]. It is important that future endeavors continue to delve into this field, with the aim of fostering a more comprehensive and nuanced understanding of the presence of mercury in the middle Araguaia floodplain.

## 5. Conclusions

The consumption of fish is an important source of protein for many people in Brazil, and many biological and ecological factors influence the amount of Hg that fish species present. The dataset assembled in this study suggests that THg contamination in fish species is a valid indicator of their trophic position in the local food web. Since Hg concentrations may differ among organisms of the same species but inhabit distinct areas along a river, it is a better variable to determine the trophic position of a fish species in its environment. Our results show that feeding habits and trophic levels are affected by regional environmental factors, which eventually control Hg bioavailability and bioaccumulation, as demonstrated here for the middle Araguaia River. 

In this analysis, the source from which Hg derives is not relevant since its presence in the environment is enough to enter the food chain and accumulate in fish species according to their feeding behavior. Geology and proximity to urbanized areas had a significant impact on biota–sediment accumulation factor values, which we believe to be due to sediment Hg constitution, food availability, and size of the food chain.

Our findings indicate that the Hg concentration in fish muscle tissue is strongly related to both body size and trophic position (FishBase). Our hypothesis was corroborated, with the trophic level and standard length explaining 94% of the variation in the THgconcentration in fish. Future studies should focus on assessing if other factors such as spatial occupation, prey availability, phylogenetic position, sex, intra and interspecific similarity, and individual preference influence the trophic position in fish.

## Figures and Tables

**Table 1:** Species and trophic guilds of fish collected in the Araguaia River floodplain in January 2019 and their respective average, minimum, and maximum THg values.

Fish Species	TP± SD ^1^	*n*	Mean ± SD THg (µg·g^−1^)	Min ^2^ THg (µg·g^−1^)	Max ^2^ THg (µg·g^−1^)
Piscivores ^3^					
*Agoniates halecinus*	2.9 ± 0.3	39	1.20 ± 0.74	0.14	2.87
*Hydrolycus tatauaia*	4.3 ± 0.8	2	0.88 ± 0.07	0.87	0.88
*Plagioscion squamosissimus*	4.4 ± 0.5	6	0.76 ± 0.34	0.53	1.44
*Raphiodon vulpinus*	4.5 ± 0.8	8	0.75 ± 0.41	0.35	1.32
*Serrasalmus rhombeus*	4.0 ± 0.4	9	0.62 ± 0.32	0.12	1.04
*Pygocentrus nattereri*	3.7 ± 0.6	31	0.58 ± 0.30	0.24	1.79
*Serrasalmus maculatus*	4.1 ± 0.7	8	0.51 ± 0.41	0.11	1.25
*Hydrolycus armatus*	4.5 ± 0.8	7	0.40 ± 0.26	0.19	0.86
Carnivores ^3^					
*Pellona castelnaeana*	3.7 ± 0.5	16	1.97 ± 1.69	0.29	6.93
*Arapaima gigas*	4.5 ± 0.0	3	0.13 ± 0.006	0.12	0.13
*Bryconops alburnoides*	3.2 ± 0.4	4	0.35 ± 0.07	0.30	0.47
*Auchenipterus nuchalis*	3.6 ± 0.5	3	0.24 ± 0.04	0.19	0.28
*Argonectes robertsi*	2.8 ± 0.4	4	0.27 ± 0.17	0.05	0.42
Omnivores ^3^					
*Triportheus elongatus*	2.9 ± 0.3	15	1.56 ± 1.01	0.19	3.18
*Serrasalmus eigenmanni*	3.7 ± 0.6	6	0.30 ± 0.19	0.09	0.62
*Metynnis hypsauchen*	3.5 ± 0.6	3	0.30 ± 0.07	0.21	0.35
*Hemiodus microlepis*	2.8 ± 0.3	5	0.26 ± 0.13	0.04	0.42
*Anodus elongatus*	3.4 ± 0.4	6	0.25 ± 0.09	0.13	0.36
*Pimelodus blochii*	3.1 ± 0.4	13	0.16 ± 0.06	0.09	0.30
Detritivores ^3^					
*Curimata inornata*	2.0 ± 0.0	31	0.17 ± 0.11	0.05	0.53
*Psectrogaster amazonica*	2.0 ± 0.0	20	0.13 ± 0.04	0.07	0.24

^1^ FishBase values for trophic position; SD: standard deviation. ^2^ Min: minimum; Max: maximum. ^3^ Trophic guild according to the FishBase.

**Table 2:** Multiple regression model for predicting the relationship between standard length (SL), trophic level, and total mercury (THg) concentration in fish species from the Araguaia River.

Predictors	Coefficients	t-Value	Significance (*p*)
ln SL	2.690 ± 0.057	45.135	<0.0001
ln med FishBase	1.114 ± 0.031	32.703	<0.0001
ln SL ∗ ln med FishBase ^1^	0.740 ± 0.023	32.842	<0.0001

^1^ the symbol (∗) denotes multiplication of the second expression by the first.

## Data Availability

The data presented in this study are available upon request from the corresponding author.
